# Oral frailty five‐item checklist to predict adverse health outcomes in community‐dwelling older adults: A Kashiwa cohort study

**DOI:** 10.1111/ggi.14634

**Published:** 2023-07-17

**Authors:** Tomoki Tanaka, Hirohiko Hirano, Kazunori Ikebe, Takayuki Ueda, Masanori Iwasaki, Maki Shirobe, Shunsuke Minakuchi, Masahiro Akishita, Hidenori Arai, Katsuya Iijima

**Affiliations:** ^1^ Institute of Gerontology The University of Tokyo Tokyo Japan; ^2^ Tokyo Metropolitan Institute for Geriatrics and Gerontology Tokyo Japan; ^3^ Department of Removable Prosthodontics and Gerodontology, Graduate School of Dentistry Osaka University Osaka Japan; ^4^ Department of Removable Prosthodontics and Gerodontology Tokyo Dental College Tokyo Japan; ^5^ Division of Preventive Dentistry, Department of Oral Health Science, Graduate School of Dental Medicine Hokkaido University Sapporo Japan; ^6^ Department of Gerodontology and Oral Rehabilitation, Graduate School of Medical and Dental Sciences Tokyo Medical and Dental University Tokyo Japan; ^7^ Department of Geriatric Medicine, Graduate School of Medicine The University of Tokyo Tokyo Japan; ^8^ National Center for Geriatrics and Gerontology Obu Japan; ^9^ Institute for Future Initiatives The University of Tokyo Tokyo Japan

**Keywords:** assessment, frailty, long‐term care, mortality, oral health

## Abstract

**Aim:**

To enable easy assessment of oral frailty; that is, an overlapping slight decline in multifaceted oral function, in any setting, we developed the oral frailty five‐item checklist (OF‐5), and examined its predictive validity for increased risks of physical frailty, physical disability and mortality among community‐dwelling older adults.

**Methods:**

This population‐based cohort study randomly selected 2044 residents in Kashiwa, Japan, with no long‐term care needs. Baseline data were collected in 2012, and follow‐up data were collected in 2013, 2014, 2016, 2018 and 2021. The OF‐5 includes five measures: fewer teeth, difficulty in chewing, difficulty in swallowing, dry mouth and low articulatory oral motor skills. Physical frailty was defined according to the Cardiovascular Health Study criteria. Physical disability and mortality determined from the long‐term care insurance receipt database were followed for 9 years.

**Results:**

Of 2031 eligible participants (mean age 73.1 ± 5.6 years; 51.1% women), 39.3% individuals with ≥2 OF‐5 points had significantly increased prevalence and new‐onset rate of physical frailty. After adjusting for potential confounders, oral frailty, defined as ≥2 OF‐5 points, was associated with increased risks of physical disability (adjusted hazard ratio 1.40; 95% confidence interval 1.14–1.72) and mortality (adjusted hazard ratio 1.44; 95% confidence interval 1.11–1.87). The highest adjusted hazard ratios were observed in older adults with coexisting physical and oral frailty.

**Conclusions:**

The OF‐5 showed strong predictive validity for physical frailty, physical disability and mortality in Japanese older adults. This assessment tool can be implemented in various settings and foster comprehensive prevention through interprofessional collaboration. **Geriatr Gerontol Int 2023; 23: 651–659**.

## Graphical abstract

To enable easy assessment of oral frailty; that is, an overlapping slight decline in multifaceted oral function, in any setting, we developed an oral frailty five‐item checklist (OF‐5), including fewer teeth, difficulties in chewing, swallowing, dry mouth and low articulatory oral motor skills. We aimed to identify the prevalence of oral frailty determined using the OF‐5, and its association with multiple adverse health outcomes among community‐dwelling older adults. The OF‐5 showed strong predictive validity for physical frailty, physical disability and mortality in Japanese older adults.

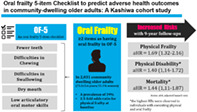

## Introduction

Strategies are required to extend healthy life expectancy and maintain independence in older adults, with frailty being crucial.^1^ Frailty is associated with decreased physical status and activity, and increased vulnerability to adverse health outcomes.^2^ Dental and oral functions in old age are attracting attention as a predictor of physical frailty and loss of independence.^3^, ^4^, ^5^, ^6^ We previously identified overlapping impairments of dental and oral functions as oral frailty that might be risk factors for physical frailty, and reported their association with adverse health outcomes, including mortality.^3^, ^4^, ^5^, ^6^, ^7^ The Japanese Society of Geriatric Dentistry has proposed oral hypofunction, which allows dental healthcare professionals to comprehensively assess, manage and intervene in the dental and oral functions of older adults.^8^ Importantly, factors related to oral frailty in old age, including nutritional status and social aspects, are complex. Hence, assessment and intervention by interprofessional work with dental healthcare professionals through various community activities and efforts for older adults are required.^8^, ^9^

For sarcopenia, a crucial factor of frailty, simple assessment items are used to enable community‐level assessment and intervention more widely.^10^, ^11^ However, oral frailty assessment items include many objective indicators that can only be assessed at limited dental institutions.^3^ Hence, a simple self‐questionnaire of oral frailty, the Oral Frailty Index‐8, has been developed to screen for oral frailty.^7^ It also included questions about oral health behaviors (tooth brushing habits and regular dental visits) and social activities, which might have broad educational implications for changing attitudes and behaviors toward a healthy lifestyle. However, a simplified assessment method other than Oral Frailty Index‐8 usable in various settings is required, including in communities. The tool should encourage interprofessional work, because Oral Frailty Index‐8 is only used for screening purposes, and high‐risk individuals are assumed to be thoroughly assessed at dental care institutions.

We developed an oral frailty five‐item checklist (OF‐5), including fewer teeth, difficulties in chewing, swallowing, dry mouth and low articulatory oral motor skills, to identify the prevalence of oral frailty and its association with multiple adverse health outcomes (physical frailty, certification of the need for long‐term care and mortality).

## Methods

### Study setting and participants

Data from a longitudinal prospective cohort study carried out in Kashiwa, Japan, aimed to identify important factors affecting healthy aging in community‐dwelling older adults, were used.^12^ Urban and rural communities intermingled in this area. In 2012, 12 000 adults aged ≥65 years without disabilities were randomly selected from the registry of Kashiwa, and invitations to participate were sent by mail. In total, 2044 older adults (1013 men and 1031 women) agreed to participate. The participants represented the regional distribution for each sex. Baseline data were collected between September and November 2012 at welfare and community centers.

Additionally, follow ups were carried out in 2013, 2014, 2016, 2018 and 2021 to examine the longitudinal association between oral frailty and new‐onset physical frailty, oral frailty and adverse health outcomes (physical disability and mortality). Participants were followed up from September 2012 to August 2021 using the receipt database from the long‐term care insurance (LTCI) service. The exclusion criteria were cognitive impairment (Mini‐Mental State Examination score <18) and missing oral frailty items and outcomes. Furthermore, we excluded participants with physical frailty at baseline examination in 2012 from the analysis of the association with new‐onset physical frailty. Figure S1 summarizes the participant selection and study flow.

This study followed the Strengthening the Reporting of Observational Studies in Epidemiology guidelines for cohort studies. The study protocol was approved by the ethics committee of the University of Tokyo (#21–192), and written informed consent was obtained from all participants. This study was carried out in accordance with the principles of the Declaration of Helsinki.

#### OF‐5

The present study defined oral frailty as “the overlap of minor declines in dental or oral functions that may increase the risk of adverse health outcomes.” OF‐5, which can be assessed in non‐dental healthcare settings and community activities, and by older people, was developed based on our previous proposal of an objective measure of oral frailty^3 7^. OF‐5 was determined using five measures (Table 1): fewer teeth, difficulty in chewing, difficulty in swallowing, dry mouth and low articulatory oral motor skills. Participants with <20 natural teeth were categorized as having fewer teeth. Difficulties in chewing, swallowing and dry mouth were assessed using questions from the Kihon Checklist.^13^ The number of teeth and articulatory oral motor skills were assessed by trained dental hygienists with experience in clinical practice under the supervision of dentists. Articulatory motor skill was assessed with oral diadochokinesis in producing the “ta” sound. Participants were asked to repetitively articulate syllables as quickly as possible for 5 s.^14^ Articulation counts were measured using a digital counter (T.K.K.3350 digital counter; Takei Scientific Instruments, Niigata, Japan). Participants with counts <6.0 times/s were categorized as having low articulatory oral motor skills.^8^ Trained dental hygienists with clinical experience carried out oral examinations under dentists' supervision. All dental staff members were trained in workshops.

**Table 1:** Oral frailty five‐item checklist

Concept	Item	Frail response
Fewer teeth	Counts by trained dental hygienists under the supervision of dentists	<20 natural teeth
Difficulty in chewing	Do you have any difficulties eating tough foods now compared with 6 months ago?	“Yes”
Difficulty in swallowing	Have you choked on your tea or soup recently?	“Yes”
Dry mouth	Do you often experience having a dry mouth?	“Yes”
Low articulatory oral motor skills	Oral diadochokinesis/ta/sound	<6.0 times/s

### Outcomes

#### Frailty

According to the Cardiovascular Health Study index criteria, physical frailty was defined based on shrinking, exhaustion, low activity, weakness and slowness.^2^, ^15^ Participants without these conditions were considered non‐frail, those with one or two conditions were considered pre‐frail and those with three or more conditions were considered physically frail. The five‐item assessment method has been reported previously.^3^, ^16^ Frailty was similarly assessed at each follow‐up survey to evaluate new‐onset frailty. The time to the first development of frailty was defined as the number of years of follow up.

#### Physical disability and mortality

Physical disability is a new requirement for LTCI. LTCI services are provided to all older adults aged ≥40 years eligible for benefits in cases of physical and/or mental disability in Japan.^17^ The Japanese LTCI Approval Board regularly investigates insured individuals' mental and physical conditions, and makes screening decisions based on a general physician's opinion. Older adults certified as dependent are subdivided into seven categories, with two requiring support and five requiring long‐term care.^17^ The present study used the public LTCI receipt database of Kashiwa City, Chiba Prefecture, Japan, to identify individuals newly certified as having a physical disability. Those independent at baseline, but later certified as requiring long‐term care (care level ≥1), were included. Independence duration was calculated from the baseline examination date to certification date. Mortality was evaluated using the LTCI system, and for those who relocated from the city, the follow‐up duration was defined until their relocation date.

### Covariates

Covariates included age; sex; body mass index; education level; living arrangement; annual income; cognitive function determined using the Mini‐Mental State Examination^18^; depressive symptoms determined using the Geriatric Depression Scale‐15^19^; exercise habits determined using the Global Physical Activity Questionnaire^20^; daily food diversity^21^; and oral supplementation, alcohol and smoking habits. Data on comorbid chronic diseases (hypertension, diabetes mellitus, dyslipidemia, osteoporosis, malignant neoplasm, stroke, chronic renal failure and heart disease) were obtained during interviews.

### Statistical analysis

All statistical analyses were carried out using spss version 29.0 (IBM Japan, Tokyo, Japan). A two‐sided *P*‐value of <0.05 was considered statistically significant.

The baseline association between OF‐5 status and physical frailty was assessed using logistic regression, and the longitudinal association between OF‐5 status at baseline and physical frailty onset, physical disability and mortality was assessed using Kaplan–Meier curves and the Cox proportional hazards model. Similarly, we examined the longitudinal association of overlapping physical and oral frailty status in the baseline survey with physical disability and mortality. Odds ratios (OR) and hazard ratios (HRs) were calculated using bivariate and multivariate models. In the multivariate model, the ratios were adjusted according to baseline status for the following continuous variables: age, body mass index, education level, cognitive function (Mini‐Mental State Examination score), depressive symptoms (Geriatric Depression Scale‐15 score) and daily food diversity score. The ratios were also adjusted for the following categorical variables: sex, living arrangement (alone/together), low annual income (≥14 or <1.4 million yen per household), exercise habits (yes/no), current alcohol habits (yes/no), current smoking habits (yes/no) and individual chronic conditions. Multiple imputation using fully conditional specifications (chained equations) was applied to impute the missing values for covariates, and 10 datasets were created. All available characteristics are listed in Table 2, and the outcome data were used for imputation.

**Table 2:** Baseline demographic and background characteristics according to oral frailty status

		Oral frailty status determined using OF‐5		
Baseline status	Overall	Non‐frail	Frail	*P* ^†^
No. participants	2031	1232 (60.7)	799 (39.3)	‐
Basic attributes				
Age (years)	73.1 (±5.6)	71.9 (±5.0)	74.9 (±6.0)	<0.001
Women	1024 (50.4)	595 (48.3%)	429 (53.7%)	0.160
Education (years)	12 (12–15)	12 (12–16)	12 (12–14)	<0.001
Living arrangements (alone)	224 (11.0)	117 (9.5%)	107 (13.4%)	0.006
Low annual income^‡^	437 (21.5)	220 (17.9%)	217 (27.2%)	<0.001
Lifestyle				
Exercise habit	1565 (77.1)	980 (79.5%)	585 (73.2%)	0.001
Drinking habit	971 (47.8)	617 (50.1%)	354 (44.3%)	0.010
Smoking habit	92 (4.5%)	56 (4.5%)	36 (4.5%)	0.906
Food diversity score	4.0 (2.0–5.0)	4.0 (2.0–5.0)	4.0 (2.0–5.0)	*0.280*
Physical and psychological attributes				
Body mass index (kg/m^2^)	22.9 ± 3.0	22.9 ± 2.9	22.8 ± 3.2	*0.612*
Cognitive function (MMSE score)	28.2 ± 1.9	28.3 (±1.8)	27.9 (±2.0)	*<0.001*
Depressive symptoms (GDS‐15 score)	2.0 (0.0–4.0)	1.0 (0.0–3.0)	3.0 (1.0–5.0)	*<0.001*
Present chronic conditions				
Hypertension	893 (44.0%)	493 (40.0%)	400 (50.1%)	<0.001
Diabetes mellitus	246 (12.1%)	134 (10.9%)	112 (14.0%)	0.033
Osteoporosis	226 (11.1%)	118 (9.6%)	108 (13.5%)	0.007
Dyslipidemia	776 (38.2%)	477 (38.7%)	299 (37.4%)	0.557
Malignant neoplasm	308 (15.2%)	161 (13.1%)	147 (18.4%)	0.001
Heart disease	370 (18.2%)	199 (16.2%)	171 (21.4%)	0.003
Chronic renal failure	15 (0.7%)	7 (0.6%)	8 (1.0%)	0.295

*Note*: Data are shown as means (±standard deviations) or medians (interquartile ranges) for quantitative measures and as the number of participants (percentages) for all qualitative measures.

^†^ The baseline differences in variables among those with and without oral frailty were analyzed using the χ^2^‐test or Fisher's exact test for categorical variables and unpaired *t*‐tests or the Mann–Whitney *U*‐test for continuous variables.

^‡^ Less than1.4 million yen per household.

Abbreviation: GDS‐15, Geriatric Depression Scale‐15; MMSE, Mini‐Mental State Examination; OF‐5, oral frailty five‐item checklist.

## Results

### Study participants

Among the 2044 participants who underwent the baseline assessment, nine were excluded due to a lack of LTCI receipt data, resulting in 2031 eligible individuals (response rate 100%, mean age 73.1 ± 5.6 years, 50.4% women) for the analysis of the cross‐sectional association with physical frailty at baseline and longitudinal association with adverse health outcomes. Additionally, 208 participants were excluded due to physical frailty at baseline (*n* = 204) or missing physical frailty items (*n* = 4). Furthermore, there were 417 absentees at all follow‐up visits. Thus, 1419 participants (response rate 77.3%, mean age 72.5 ± 5.4 years, 50.2% women) were eligible for assessment of the longitudinal association between OF‐5 status and physical frailty onset (Fig. S1).

### Baseline characteristics and oral frailty determined using OF‐5

Table 2 presents the basic characteristics of the study participants, including those with oral frailty. Older adults with oral frailty were more advanced in age, tended to be women (*P* = 0.16), had shorter educational backgrounds, lived alone, had low annual income, had lower likelihood of exercise or drinking habits, and showed higher depressive tendency scores and slightly lower cognitive function. Furthermore, individuals with oral frailty had a history of hypertension, diabetes, osteoporosis, malignant neoplasms and heart disease, although no notable differences were observed in smoking habits, food diversity score, body mass index, dyslipidemia or chronic renal failure history compared with those without oral frailty.

### OF‐5 and physical frailty

The cross‐sectional and longitudinal associations between OF‐5 and physical frailty are shown in Table 3. At baseline, 204 (10%) of 2031 patients had physical frailty, and 274 (19.3%) of 1419 patients had new‐onset frailty at follow up. After adjustment in the multivariate model, the four items of OF‐5 were significantly associated with higher ORs and HRs for frailty, except “fewer teeth.” In addition, the association between the number of overlaps in these five OF‐5 items and physical frailty was significant only when two or more items were applicable in both cross‐sectional and longitudinal analyses. Therefore, we defined individuals with two or more items as having oral frailty in OF‐5. Of the 2031 participants, 799 (39.3%) belonged to the oral frailty category, as classified by OF‐5. Older adults with oral frailty using OF‐5 had significantly increased ORs for physical frailty at baseline, and significantly higher HRs for new‐onset physical frailty.

**Table 3:** Cross‐sectional and longitudinal associations of oral frailty five‐item checklist with physical frailty

Baseline status	Cross‐sectional analysis: physical frailty				Longitudinal analysis: physical frailty onset			
	*n*	No. cases (%)	Unadjusted OR (95% CI)	Adjusted OR (95% CI)^‡^	*n*	No. cases (%)	Unadjusted HR (95% CI)	Adjusted HR (95% CI)^‡^
Overall	2031	204 (10.0)			1419	274 (19.3)		
Item of oral frailty								
Fewer teeth	646	96 (14.9)	2.06 (1.54–2.77)^†^	1.11 (0.79–1.54)	419	90 (21.5)	1.41 (1.10–1.82)^†^	1.03 (0.78–1.34)
Difficulty in chewing	321	70 (21.8)	3.28 (2.39–4.51)^†^	2.13 (1.68–3.52)^†^	199	56 (28.1)	1.76 (1.31–2.36)^†^	1.43 (1.06–1.95)^†^
Difficulty in swallowing	392	67 (17.1)	2.26 (1.65–3.10)^†^	1.87 (1.31–2.69)^†^	263	74 (28.1)	1.78 (1.36–2.32)^†^	1.53 (1.16–2.01)^†^
Dry mouth	563	90 (16.0)	2.26 (1.68–3.04)^†^	1.99 (1.42–2.79)^†^	383	105 (27.4)	1.90 (1.49–2.42)^†^	1.57 (1.12–2.01)^†^
Low articulatory oral motor skills	809	118 (14.6)	2.26 (1.68–3.03)^†^	1.58 (1.13–2.21)^†^	522	122 (23.4)	1.54 (1.22–1.96)^†^	1.22 (1.01–1.59)^†^
OF‐5 score								
0	554	23 (4.2)	1.00 (reference)	1.00 (reference)	405	48 (11.9)	1.00 (reference)	1.00 (reference)
1	678	43 (6.3)	1.56 (0.83–2.63)	0.98 (0.56–1.71)	502	92 (18.3)	1.74 (1.23–2.47)^†^	1.45 (0.81–2.11)
2	471	60 (12.7)	3.37 (2.05–5.54)^†^	1.78 (1.04–3.04)^†^	320	74 (23.1)	2.44 (1.70–3.51)^†^	1.78 (1.22–2.60)^†^
3	221	42 (19.0)	5.42 (2.08–9.23)^†^	2.17 (1.20–3.93)^†^	136	39 (28.7)	3.43 (2.25–5.23)^†^	2.26 (1.46–3.51)^†^
4	87	28 (32.2)	11.0 (5.93–20.2)^†^	4.34 (2.17–8.68)^†^	44	15 (34.1)	3.70 (2.07–6.61)^†^	2.22 (1.17–4.20)^†^
5	20	8 (40.0)	15.4 (5.74–41.3)^†^	5.63 (1.87–17.0)^†^	12	6 (50.0)	5.67 (2.43–13.3)^†^	3.26 (1.34–7.93)^†^
Oral frailty states								
Oral non‐frailty, 0–1 OF‐5 score	1232	66 (5.4)	1.00 (reference)	1.00 (reference)	907	140 (15.4)	1.00 (reference)	1.00 (reference)
Oral frailty, ≥2 OF‐5 score	799	138 (17.3)	3.69 (2.71–5.02)^†^	2.45 (1.74–3.44)^†^	512	134 (26.2)	2.06 (1.63–2.62)^†^	1.69 (1.32–2.16)^†^

^†^ Statistically significant (*P* < 0.050).

^‡^ Hazard ratios were adjusted by potentially confounding baseline factors as follows: age, sex, body mass index, education level, living alone, low annual income, cognitive function, exercise habit, daily food diversity, drinking habit, smoking habit and chronic diseases.

Abbreviation: CI, confidence interval; HR, hazard ratio; ODK, oral diadochokinesis; OF‐5, oral frailty five‐item checklist; OR, odds ratio.

### Oral frailty and adverse health outcomes

**Figure 1:**
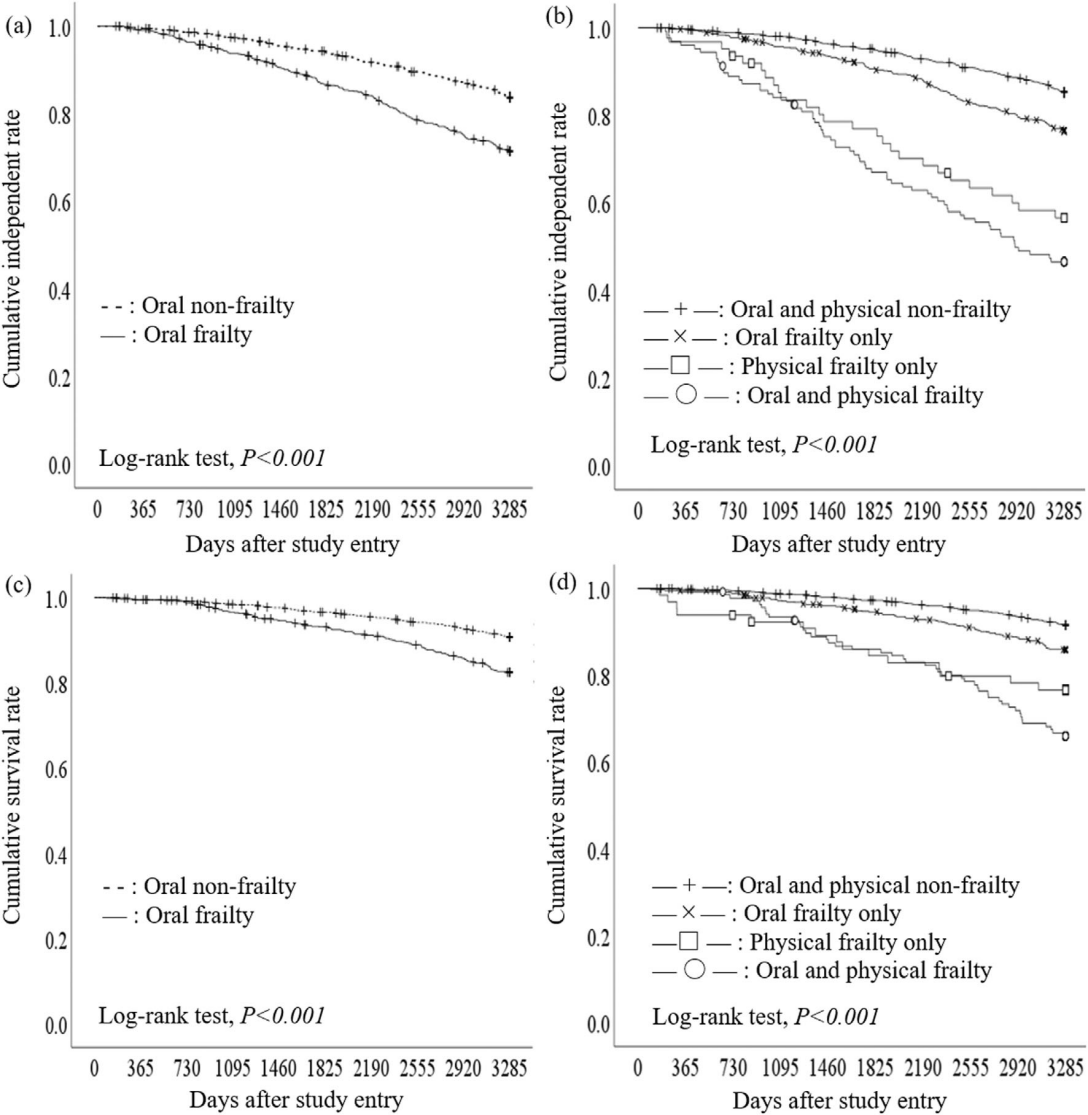
Kaplan–Meier curves for (a, b) physical disability and (c, d) mortality stratified by oral frailty status. Kaplan–Meier curves for physical disability stratified by (a) oral frailty status and (b) oral and physical frailty status at baseline. Kaplan–Meier curves for mortality stratified by (c) oral frailty status and (d) oral and physical frailty status at baseline.

Analyzing the association between OF‐5‐identified oral frailty and physical disability and mortality over 9 years, considering baseline physical frailty, showed that 400 (19.7%) individuals developed functional disability and 248 (12.2%) died during the follow‐up period. Figure 1 shows the Kaplan–Meier curves for disability and mortality with and without (i) oral frailty and (ii) oral frailty and physical frailty. Older adults with oral frailty were significantly more likely to experience disability and die, even when adjusted for multivariate models (Table 4). Increased HRs for physical disability or mortality were also observed in older adults having oral frailty without physical frailty. Furthermore, the highest HRs were observed in patients with coexisting physical and oral frailty, showing that oral frailty increased the HR for adverse health outcomes, even among older adults with physical frailty.

**Table 4:** Longitudinal association of oral frailty with physical disability and all‐cause mortality

Baseline status	Physical disability^†^				Mortality			
	*n*	No. cases (%)	Unadjusted HR (95% CI)	Adjusted HR (95% CI)^‡^	*n*	No. cases (%)	Unadjusted HR (95% CI)	Adjusted HR (95% CI)^§^
Overall	1947	400 (20.5)			2031	248 (12.2)		
Oral frailty								
Oral non‐frailty	1191	189 (15.9)	1.00 (reference)	1.00 (reference)	1232	111 (9.0)	1.00 (reference)	1.00 (reference)
Oral frailty	756	211 (27.9)	1.92 (1.58–2.33)^†^	1.40 (1.14–1.72)^†^	799	137 (17.1)	2.00 (1.56–2.57)^†^	1.44 (1.11–1.87)^†^
Oral frailty × physical frailty								
Oral and physical non‐frail	1129	163 (14.4)	1.00 (reference)	1.00 (reference)	1166	96 (8.2)	1.00 (reference)	1.00 (reference)
Oral frailty only	630	145 (23.0)	1.69 (1.35–2.11)^†^	1.32 (1.05–1.66)^†^	661	91 (13.8)	1.73 (1.30–2.31)^†^	1.35 (1.01–1.82)^†^
Physical frailty only	62	26 (41.0)	3.69 (2.44–5.58)^†^	2.22 (1.43–3.44)^†^	66	15 (22.7)	3.15 (1.83–5.42)^†^	2.22 (1.25–3.94)^†^
Oral and physical frailty	126	66 (52.4)	5.09 (3.82–6.77)^†^	2.87 (2.03–3.82)^†^	138	46 (33.3)	4.73 (3.33–6.73)^†^	2.79 (1.87–4.16)^†^

^†^ Statistically significant (*P* < 0.050).

^‡^ A total of 84 older adults who died before receiving long‐term care certification were excluded from the analysis.

^§^ Hazard ratios were adjusted for potentially confounding baseline factors as follows: age, sex, body mass index, education level, living alone, low annual income, cognitive function, exercise habit, daily food diversity, alcohol habit, smoking habit and chronic diseases.

Abbreviation: CI, confidence interval; HR, hazard ratio.

## Discussion

Oral frailty refers to the concurrent minor declines in dental and oral functions that can contribute to adverse health outcomes. The OF‐5, a practical checklist for oral frailty, was developed to enable assessment in clinical settings, community activities and self‐evaluation by older adults. We evaluated the predictive ability of OF‐5 for adverse health outcomes in community‐dwelling older adults. The findings showed a high prevalence of oral frailty among this population, with significantly higher rates of physical frailty and new‐onset rates. Furthermore, individuals with new‐onset oral frailty had increased HRs for physical disability and mortality, despite adjusting for frailty.

OF‐5 includes self‐administered questionnaires to evaluate difficulties with chewing, swallowing and dry mouth, part of the widely used Kihon Checklist in Japan for long‐term care prevention and frailty evaluation.^13^, ^22^, ^23^ Additionally, difficulties in chewing and swallowing were included in the 15‐question Questionnaire for Older Adults used in the integrated implementation of health and care prevention projects for older adults newly enacted in 2020. Hence, they are also evaluated during the health examinations of older adults.^24^ Furthermore, each of these three items has been associated with frailty, prediction of the need for care and mortality, consistent with the present results.^3^, ^22^, ^25^ Therefore, it is reasonable to include these three self‐administered questionnaires in OF‐5 because of their generalizability and predictive validity for physical outcomes.

OF‐5 comprised two objective indicators, namely, “having fewer than 20 teeth” and “articulatory oral motor skills assessed by oral diadochokinesis.” Although univariate analysis showed an association between the number of teeth and frailty, no significant association was observed after adjusting for age and other factors. Nevertheless, chewing difficulty was significantly associated with increased ratios for outcomes, suggesting that denture maintenance through regular dental visits might be important. Furthermore, in Japan, the correlation between fewer teeth and a heightened risk of adverse health outcomes has been extensively researched.^26^, ^27^ Considering the significance of the number of teeth for oral function, incorporating it as a new item in OF‐5 is appropriate.

Although dental healthcare professionals evaluated the number of teeth, community healthcare providers might not have the required expertise. Thus, developing a self‐report questionnaire that older adults can complete is necessary. Although the self‐reported number of teeth is widely used in Japan and its validity has been addressed previously, further in‐depth surveys of older adults are warranted.^28^, ^29^

As aforementioned, OF‐5 includes articulation and oral motor skills assessed by oral diadochokinesis/ta/sound.^14^ Oral function comprises diverse factors; this oral motor skill is an important factor that the other four items cannot represent.^8^ Furthermore, oral diadochokinesis is associated with various physical outcomes, and improvement is easily perceived through interventions.^30^ Hence, it is also expected to promote awareness and behavioral change among older adults.^3^, ^30^, ^31^, ^32^ Oral diadochokinesis can be evaluated without equipment or smartphone applications, making it accessible in community healthcare settings and activities led by residents. Therefore, it is reasonable and essential to include oral diadochokinesis as one of the five new items in OF‐5. Overall, the present study findings are not sample‐specific, but generalizable, supported by similarities with previous studies that showed associations between each of the five new items and physical outcomes. OF‐5 identified a 39% prevalence of oral frailty in community‐dwelling older adults, with a notably higher HR for physical disability and mortality. This association was stronger among older adults without physical frailty. However, even those with physical frailty showed an elevated HR if oral frailty was present. As physical frailty had a prevalence of 10% in the present study, detecting oral frailty at an early stage is crucial, as it might occur before physical frailty. Addressing oral frailty in community activities and healthcare settings, and involving dental care might reduce the risk of frailty and its adverse outcomes. In Japan, oral hypofunction has been proposed, and oral function management has been covered by insurance in community dental care settings.^8^ Thus, a system for delivering dental interventions has been established. However, future studies on an alternative indicator comprising a self‐administered questionnaire are required. Oral frailty determined by OF‐5 can be evaluated in community activities and by older adults, even without a dental care system, such as oral hypofunction assessments.

The study strengths were its large sample size and extended follow‐up period, resulting in a high follow‐up rate for adverse health outcomes. However, certain limitations were applicable. Although multivariate modeling adjusted for several confounding factors, unmeasured confounding variables could have influenced the findings. Additionally, despite randomized sampling of community‐dwelling older adults, the healthy volunteer effect might have influenced the representativeness of the population. The inability to follow up on frailty development in older adults who missed all follow‐up visits might have led to the underestimation of frailty incidence due to survival bias. Finally, as this study was a longitudinal cohort study, identifying causal relationships requires future randomized controlled trials.

The present study introduces a new assessment tool for oral frailty, OF‐5, suitable for clinical and community settings. Among community‐dwelling older adults, OF‐5 identified a prevalence of 39% for oral frailty, which correlated with higher rates of physical frailty and increased HRs for physical disability and mortality. These data show that new‐onset oral frailty also has implications for the onset of frailty and adverse health outcomes in community‐dwelling older adults, and is particularly urgent for older adults with physical frailty.

## Disclosure statement

The authors declare no conflict of interest.

## Supporting information


**Figure S1.** Study diagram of the participants. LTCI, long‐term care insurance.

## Data Availability

The data that support the findings of this study are available on request from the corresponding author. The data are not publicly available due to privacy or ethical restrictions.
