# Supplementary figures and images for: Oral frailty five‐item checklist to predict adverse health outcomes in community‐dwelling older adults: A Kashiwa cohort study
Source: Geriatr Gerontol Int. 2023 Jul 17;23(9):651–9. doi: 10.1111/ggi.14634 (PMC11503571; doi:10.1111/ggi.14634)

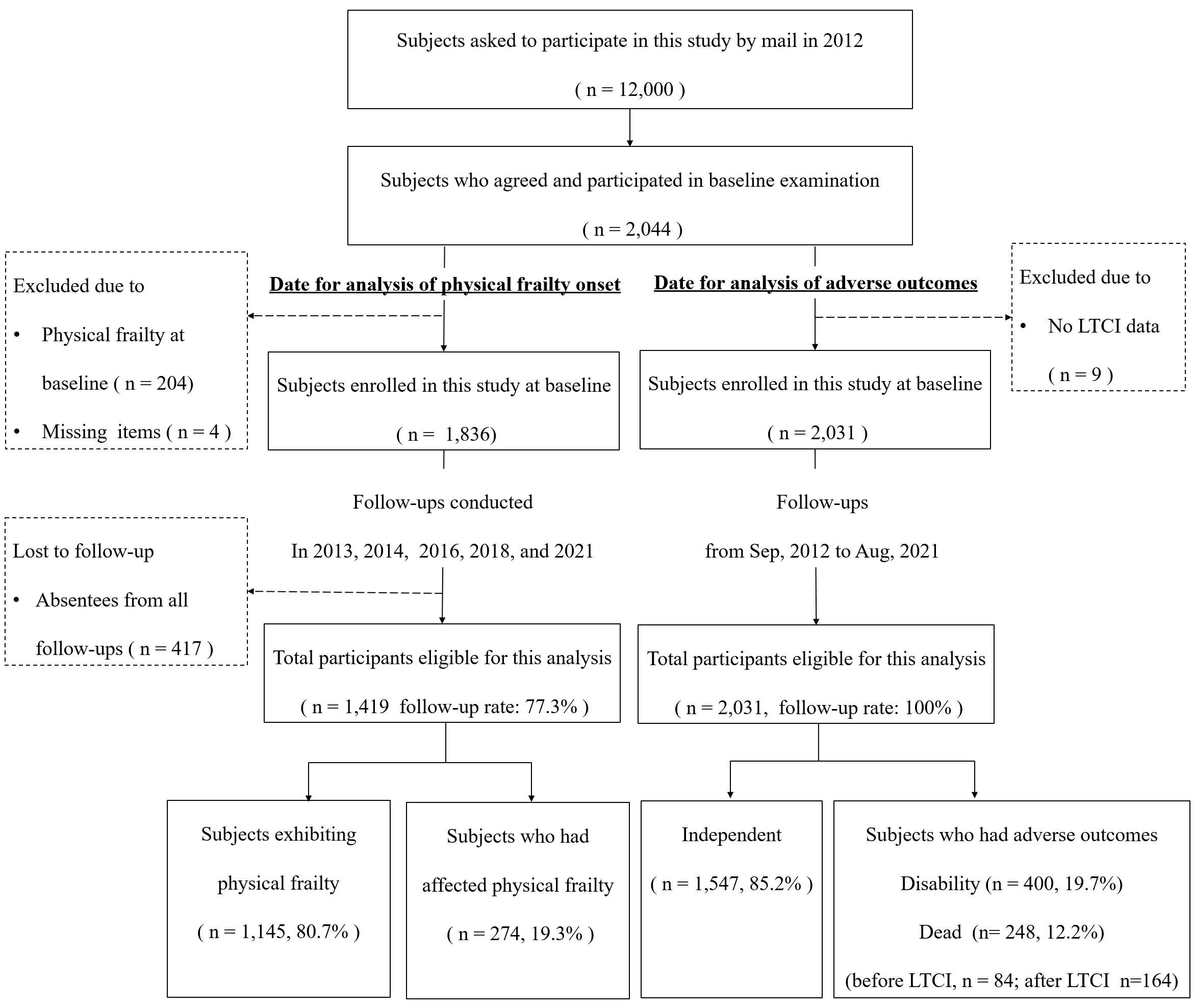

Supplement: Supplementary file 1 — Figure S1. Study diagram of the participants. LTCI, long‐term care insurance. [file GGI-23-651-s001.tif]
